# The Different Clinical Features Between Autoimmune and Infectious Status Epilepticus

**DOI:** 10.3389/fneur.2019.00025

**Published:** 2019-02-13

**Authors:** Chih-Hsiang Lin, Yan-Ting Lu, Chen-Jui Ho, Fu-Yuan Shih, Meng-Han Tsai

**Affiliations:** ^1^Department of Neurology, Kaohsiung Chang Gung Memorial Hospital, College of Medicine, Chang Gung University, Kaohsiung, Taiwan; ^2^Department of Neurosurgery, Kaohsiung Chang Gung Memorial Hospital, College of Medicine, Chang Gung University, Kaohsiung, Taiwan

**Keywords:** status epilepticus, inflammatory, autoimmune, infection, autoantibody

## Abstract

**Objective:** The prognosis of status epilepticus (SE) is highly related to the underlying etiology. Inflammation of the central nervous system (CNS), including infection and autoimmune encephalitis, is one of the treatable conditions causing SE. The initial presentation of infectious and autoimmune CNS disorders can be quite similar, which may be difficult to differentiate at the beginning. However, treatment for these entities can be quite different. In this study, we aim to identify the differences in clinical features among patients with infectious and autoimmune SE, which could help the clinicians to select initial investigation and ensuing therapies that may improve overall outcomes.

**Methods:** This was a retrospective study that included 501 patients with SE within a period of 10.5-years. Patients with inflammatory etiology were collected and separated into infectious and autoimmune SE. The symptoms at onset, SE semiology, status epilepticus severity score, and END-IT score at admission, treatment for SE, and outcome (modified Rankin Scale) on discharge and last follow-up were recorded. Data on the first cerebrospinal fluid, electroencephalography, and magnetic resonance imaging were also collected.

**Results:** Forty-six (9.2%) of the 501 patients had SE with inflammatory etiology. Twenty-five (5%) patients were autoimmune SE and 21 (4.2%) were infectious SE. Patients with autoimmune SE have younger age and female predominance. As for clinical presentations, psychosis, non-convulsive SE, and super refractory SE were more common in patients with autoimmune SE. Nevertheless, the prognosis showed no difference between the two groups.

**Conclusion:** The different initial clinical presentations and patient characteristics may provide some clues about the underlying etiology of SE. When inflammatory etiology is suspected in patients with SE, younger age, female sex, psychosis, non-convulsive SE, and super refractory SE are clinical features that suggest an autoimmune etiology.

## Introduction

Status epilepticus (SE) is a neurological emergency associated with significant morbidity and mortality that usually requires admission to an intensive care unit (1–3). The goal when treating SE is to terminate the clinical and electrographic seizure activities as soon as possible (4). Even though antiepileptic drugs (AEDs) can be used to control seizures (5), the prognosis of SE is highly related to age and the underlying etiology (6–8). To further improve outcomes, targeted management of the underlying causes may be required (4, 9).

Brain inflammation can also cause SE (10, 11), including central nervous system (CNS) infections and autoimmune encephalitis (12). These conditions can be treated and may result in significantly different outcomes (13–15). Altered mental status is the most common initial presentation of inflammatory SE (12). However, it is an ambiguous sign that provides little information on the underlying etiology. Currently available investigations could help in initial differential diagnosis but have some limitations. Laboratory tests such as bacterial or viral culture, polymerase chain reaction (PCR) for specific pathogens, or autoantibody testing may not be immediately available (13, 16) and the results may take a few days or weeks to return. Cerebrospinal fluid (CSF) studies are useful to confirm the diagnose of bacterial infections, but are less effective in distinguishing between viral infections and autoimmune processes (13, 17). Magnetic resonance imaging (MRI) can provide evidence of CNS inflammation, but not the underlying cause of the inflammation (18). Electroencephalography (EEG) may sometimes show patterns that suggest a specific diagnosis, such as extreme delta brush in patients with anti-N-methyl-D-aspartate (NMDA) receptor encephalitis, but the findings are mostly non-specific (19).

Only two studies have specifically addressed the differences between infectious and autoimmune etiology. Spatola et al. were the first to report that patients with an infectious etiology were older in age and had a more severe clinical presentation at first encounter (20). Subsequently, Shin et al. found that patients with an autoimmune etiology were younger (11). Herein, we retrospectively reviewed our patients with SE and an inflammatory etiology over a 10.5-year period. We aimed to identify the presenting factors that may assist clinicians in differentiating the two entities earlier, which may lead to faster targeted treatment and better patient outcomes.

## Materials and Methods

### Study Design

We retrospectively reviewed the medical records of all patients with SE admitted to the Neurological Intensive Care Unit at Kaohsiung Chang Gung Memorial Hospital between January 2006 and July 2016. This study was approved by the Chang Gung Medical Foundation Institutional Review Board.

### Definitions and Criteria

SE was defined as 5 min or more of continuous clinical and/or electrographic seizure activity or recurrent seizure activity without recovery (returning to baseline) between seizures (21). Refractory SE was defined as SE not responded to first-line therapy (benzodiazepine) or second-line therapy and requiring general anesthesia (22). Super refractory SE was defined as SE continues 24 h or more after the onset of anesthesia, including those cases in which the SE recurs on the reduction or withdrawal of anesthesia (22). The semiology and etiology of SE were classified according to the International League Against Epilepsy Task Force report (23).

Inflammatory SE was defined as SE due to acute inflammation of the brain parenchyma, with or without the involvement of the meninges (12), and further divided into SE due to CNS infection and autoimmune SE. Autoimmune SE included autoimmune encephalitis and systemic autoimmune disorders causing SE (23). Patients with an identified etiology for SE such as cerebrovascular disease, intracranial tumor, head trauma, metabolic disturbance, alcohol-related, AED withdrawal, neurodegenerative disease, mitochondrial disease, and medically refractory epilepsy were excluded. Patients with an unknown etiology and those without CSF data were also excluded from this study.

Autoimmune SE was defined as suggested by previous experts' consensus (16):

1. Subacute onset (rapid progression of fewer than 3 months) of working memory deficits (short-term memory loss), altered mental status, or psychiatric symptoms.
2. At least one of the followings:
  - New focal CNS findings
  - Seizures not explained by a previously known seizure disorder
  - CSF pleocytosis (white blood cell count of more than five cells per mm3)
  - MRI features suggestive of encephalitis
3. Reasonable exclusion of alternative causes

SE patients who had positive neuronal surface auto-antibodies testing (EUROIMMUN, Autoimmune Encephalitis Mosaic 6 assay, Germany) in serum or CSF were also considered as autoimmune SE.

Infectious SE was diagnosed if microbiologic studies demonstrated an infectious agent. Those without evidence of microbiologic studies would have to fulfill one of the underlying criteria (20): (1) fever>38.5°C, (2) increased white blood cell count or C-reactive protein, (3) findings highly suggestive of a bacterial infection, such as turbid CSF, neutrophilic pleocytosis, or low CSF to serum glucose ratio (<0.5), or (4) clinical picture suggestive of a viral origin plus lymphocytic pleocytosis on CSF study with positive PCR result or serology test shows a 4-fold increase of viral antibodies 3 weeks after the onset of illness (24).

Clinical information was recorded using a standardized evaluation form, including the symptoms at onset, SE semiology and classification, status epilepticus severity score (STESS) (25) and the END-IT score (26) at admission, treatment for SE, and outcome at discharge and last follow-up. A STESS score ≥3 (25) or an END-IT score ≥3 (26) suggested a poor outcome. Data on the first acquired CSF, EEG, and MRI studies were collected. The EEG was described according to the 2012 American Clinical Neurophysiology Society's (ACNS) Standardized Critical Care EEG Terminology (27), which we categorized into background slowing activity, sporadic epileptiform discharge, periodic discharge, and electrographic seizures (11). MRI findings including the location and symmetry of signal changes on fluid-attenuated inversion recovery (FLAIR) and diffusion-weighted imaging (DWI) were recorded (10). Clinical outcomes at discharge and the last follow-up were graded using the modified Rankin Scale (mRS). A good outcome was defined as an mRS score <3 and a poor outcome was defined as an mRS score ≧3.

### Statistical Analysis

Statistical analyses were performed using the IBM SPSS Statistics for Windows (version 22; IBM Corp., Armonk, NY, United States). To compare demographic data between infectious and autoimmune groups, categorical variables were assessed using Chi-square or Fisher exact tests, and continuous variables were compared using the Mann-Whitney *U*-test. *p* < 0.05 was considered as statistically significant.

## Results

**Figure 1:**
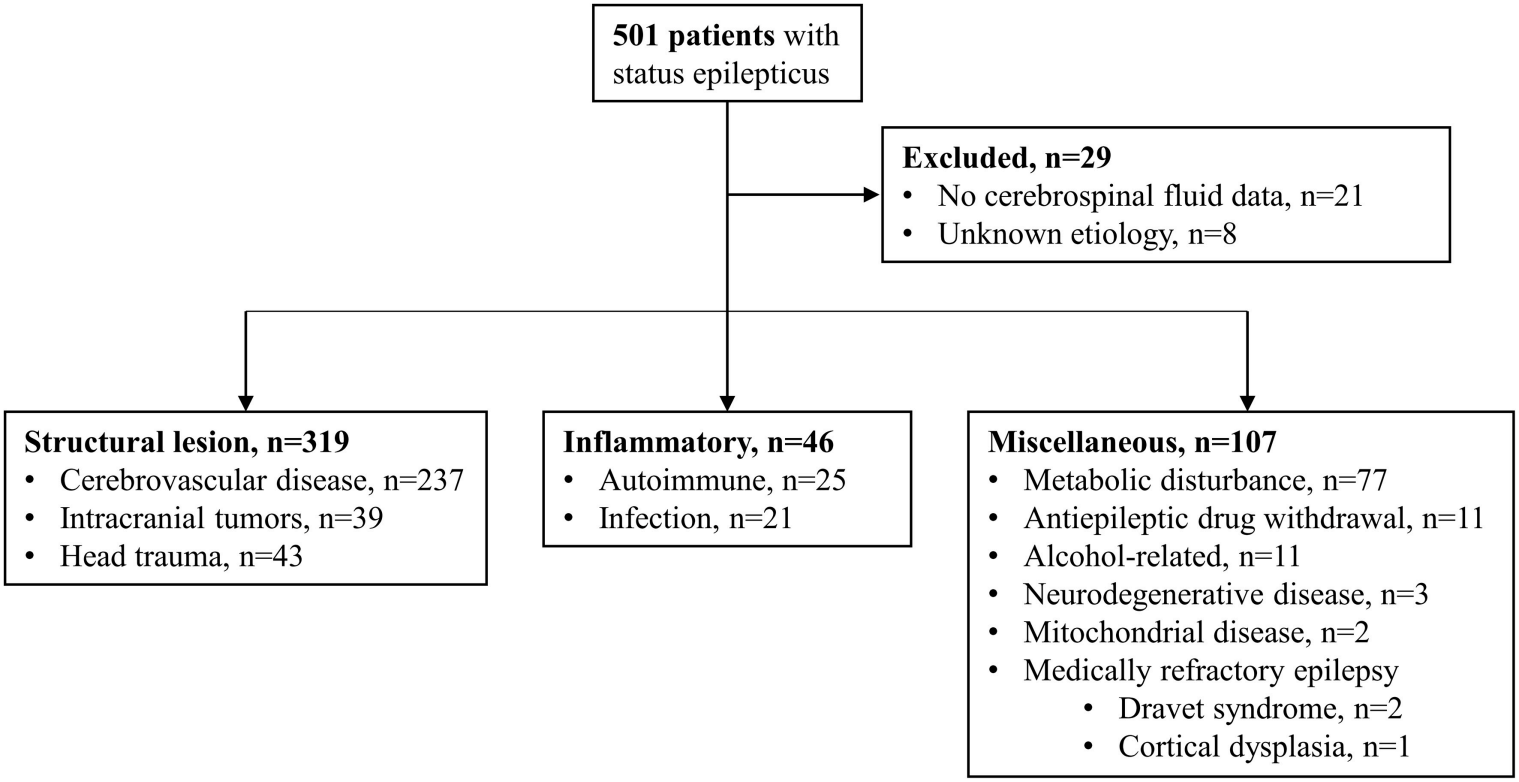
Study population and etiologies of status epilepticus.

During the 10.5-year study period (January 2006–June 2016), 501 patients with SE were reviewed, of whom 46 (9.2%) had an inflammatory etiology, including 25 females (54.3%) and 21 males (45.7%). Of the excluded patients, 237 had cerebrovascular disease, 77 had metabolic disturbances, 43 had head trauma, 39 had intracranial tumors, 11 had AED withdrawal, 11 had alcohol-related SE, three had neurodegenerative diseases, two had mitochondrial diseases, and three had medically refractory epilepsy. Of the three patients with medically refractory epilepsy, two had Dravet syndrome and one had focal cortical dysplasia. Patients without CSF data (*n* = 21) and those with an unknown etiology (*n* = 8) were also excluded (Figure 1).

The clinical characteristics of the 46 patients with inflammatory SE are presented in Table 1. Among the 46 patients, 25 (54.3%) had autoimmune SE, and 21 (45.7%) had infectious SE. In the patients with autoimmune SE, five were related to anti-NMDA receptor encephalitis, four were related to Hashimoto encephalopathy, one was related to CNS lupus, one was related to anti-collapsin response mediator protein 5 encephalitis, and 14 were diagnosed according to the criteria of autoimmune encephalitis (16). Of these 14 patients, five had received cell-based anti-neuronal antibody assays with negative results. The remaining nine patients did not receive anti-neuronal auto-antibody tests as the test was not available at the time of diagnosis. With regards to the patients with infectious SE, six had bacterial infections, 12 had viral infections, two had cryptococcal meningitis, and one had Creutzfeldt-Jakob disease.

**Table 1:** Demographic data of inflammatory SE patients.

	**Patients (*n* = 46)**
Onset age (years)	45 (28–60)
Female	25 (54.3)
Onset symptom	
Fever	24 (52.2)
Decreased consciousness	17 (37.0)
Seizure	10 (21.7)
Upper respiratory tract infection	8 (17.4)
Headache	6 (13.0)
Psychosis	6 (13.0)
Fatigue	3 (6.5)
Cognitive decline	1 (2.2)
Latency of seizure after onset symptoms (days)	3 (0–7)
STESS ≥3 at admission	18 (39.1)
**END-IT score ≥3 at admission**	**43 (93.5)**
SE with prominent motor symptoms	37 (80.4)
Generalized convulsive SE	25 (54.3)
Epilepsia partialis continua	6 (13.0)
Focal onset evolving into bilateral convulsive SE	4 (8.7)
Myoclonic SE with coma	1 (2.2)
Hyperkinetic SE	1 (2.2)
Non-convulsive SE	9 (19.6)
Non-convulsive SE with coma	6 (13.0)
Myoclonic absence status	1 (2.2)
Non-convulsive SE without impairment of consciousness	1 (2.2)
Aphasic status	1 (2.2)
**Number of AEDs used**	**3 (2-3)**
**Refractory SE**	**36 (78.3)**
**Super refractory SE**	**19 (41.3)**
Required general anesthesia for SE control	21 (45.7)
Death during admission	13 (28.3)
Days of admission	39 (26-79)
Days in ICU	33.5 (11-60)
More than two AEDs at discharge	17 (36.9)
Good outcome at discharge (mRS<3)	17 (36.9)
Good outcome at last follow up (mRS<3)	16 (34.8)

Continuous variables were presented as median (interquartile range)

*Categorical variables were presented as n (%)*.

*AED, antiepileptic drug; ICU, intensive care unit; mRS, modified Rankin Scale; SE, status epilepticus; STESS, status epilepticus severity score*.

The clinical features of infectious and autoimmune SE are compared in Table 2. The median age at onset of the patients with autoimmune SE was younger than that of the patients with infectious SE (32 vs. 56, *p* = 0.015), and more of the patients with autoimmune SE were female compared to those with infectious SE (68.0 vs. 38.1%, *p* = 0.043). The initial presentation of both groups was similar, including the STESS and END-IT score at admission, onset symptoms, and latency of seizures after the initial symptoms. Psychosis was the presenting symptom only in the autoimmune SE group (24.0 vs. 0.0%, *p* = 0.025) and non-convulsive SE was more prevalent among the patients with autoimmune SE compared to those with infectious SE (32.0 vs. 4.8%, *p* = 0.027). Refractory SE occurred more commonly in the autoimmune SE than in the infectious SE group, but the difference was not statistically significant (88.0 vs. 66.7%, *p* = 0.081). Super refractory SE was more common in the autoimmune SE group than in the infectious SE group (41.3 vs. 19.0%, *p* = 0.007). The number of AEDs used was similar between both groups, but the use of general anesthesia was more common in the autoimmune SE group than in the infectious SE group (64.0 vs. 23.8%, *p* = 0.006). However, the duration of admission or ICU stay, mRS score at discharge, and mortality rate during admission were similar between the two groups. The sensitivity and specificity for STESS to predict the outcome at discharge were 70.6 and 44.8%, respectively, compared to 68.8 and 45.5% at last follow-up. The sensitivity and specificity for the END-IT score to predict the outcome at discharge were 9.4 and 100.0%, respectively, compared to 21.4 and 100.0% at last follow-up.

**Table 2:** Comparison of the clinical features of autoimmune and infectious SE.

	**Autoimmune SE (*n* = 25)**	**Infectious SE (*n* = 21)**	***p*-value**	**OR (95% CI)**
Onset age (years)	32 (23–49.5)	56 (36.5–68.5)	0.015	
Female	17 (68.0)	8 (38.1)	0.043	0.29 (0.09–0.98)
Onset symptom				
Fever	13 (52.0)	11 (52.4)	0.979	0.99 (0.31–3.15)
Decrease consciousness	9 (36.0)	8 (38.1)	0.883	0.91 (0.28–3.04)
Seizure	6 (24.0)	4 (19.0)	0.685	1.34 (0.32–5.58)
Upper respiratory tract infection	5 (20.0)	3 (14.3)	0.611	1.50 (0.31–7.19)
Headache	2 (8.0)	4 (19.0)	0.268	0.37 (0.06–2.26)
Psychosis	6 (24.0)	0 (0.0)	0.025	
Fatigue	1 (4.0)	2 (9.5)	0.450	0.4 (0.03–4.70)
Cognitive decline	0 (0.0)	1 (4.8)	0.806	0.41 (0.01–11.68)
Latency of seizure after onset symptoms (days)	3 (0–7)	2 (0–8.5)	0.892	
STESS ≥3 at admission	11 (44.0)	7 (33.3)	0.460	1.57 (0.47–5.23)
**END-IT score ≥3 at admission**	**23 (92.0)**	**20 (95.2)**	**1.000**	**1.74 (0.15–20.65)**
SE with prominent motor symptoms	17 (68.0)	20 (95.2)	0.027	0.11 (0.01–0.94)
Generalized convulsive SE	11	14		
Epilepsia partialis continua	3	3		
Focal onset evolving into bilateral convulsive SE	2	2		
Myoclonic SE with coma	0	1		
Hyperkinetic SE	1	0		
Non-convulsive SE	8 (32.0)	1 (4.8)	0.027	9.41 (1.07–83.01)
Non-convulsive SE with coma	5	1		
Myoclonic absence status	1	0		
Non-convulsive SE without impairment of consciousness	1	0		
Aphasic status	1	0		
**Number of AED used**	**3 (2–3)**	**3 (1–3)**	**0.159**	
**Refractory SE**	**22 (88.0)**	**14 (66.7)**	**0.081**	**3.67(0.81–16.59)**
**Super refractory SE**	**15 (41.3)**	**4 (19.0)**	**0.007**	**6.38 (1.65–24.63)**
Required general anesthesia for SE control	16 (64.0)	5 (23.8)	0.006	5.69 (1.56–20.76)
Death during admission	5 (20.0)	8 (38.1)	0.175	0.41 (0.11–1.52)
Days of admission	40 (21–91)	33 (26–77.5)	0.817	
Days in ICU	34 (10.5-63.5)	33 (15–57)	0.869	
More than two AEDs at discharge	11 (44.0)	6 (28.6)	0.280	1.96 (0.57–6.74)
Good prognosis at discharge (mRS<3)	11 (44.0)	6 (28.6)	0.280	1.96 (0.57–6.74)
Good prognosis at last follow up (mRS<3)	10 (66.7)	6 (50.0)	0.381	2.00 (0.42–9.52)

*Continuous variables were presented as median (interquartile range)*.

*Categorical variables were presented as n (%)*.

*AED, antiepileptic drug; CI, confidence interval; ICU, intensive care unit; mRS, modified Rankin Scale; OR, odds ratio; SE, status epilepticus; STESS, status epilepticus severity score*.

The results of CSF and EEG are presented in Table 3 and the MRI findings are summarized in Table 4. Patients with infectious SE had a higher median CSF protein level (93.0 mg/dL vs. 34.8 mg/dL, *p* = 0.014), higher median white blood cell count (20 vs. 3 cell/mm^3^, *p* = 0.011), higher percentage of neutrophilic predominance (52.9 vs. 15.4%, *p* = 0.034), and higher percentage of low CSF/blood glucose ratio (56.3 vs. 24.0%, *p* = 0.036) compared to the patients with autoimmune SE, who had a higher percentage of lymphocytic predominance (84.6 vs. 47.1%, *p* = 0.034). There was no significant difference in IgG index between the two groups. The autoimmune SE group tended to have a higher rate of background slowing activity in the first EEG, but the difference between autoimmune and infection was not statistically different (56.0 vs. 28.6%, *p* = 0.081). The presence of sporadic epileptiform discharge, periodic discharge, or electrographic seizure was similar among the two groups in the first EEG study. With regards to the first MRI findings, an abnormal FLAIR signal was observed in 11 patients with autoimmune SE and eight patients with infectious SE. A restricted diffusion signal on DWI was found in 14 patients with autoimmune SE and 10 patients with infectious SE. However, there were no significant differences in abnormalities in the FLAIR and DWI signals between the two groups. Detailed descriptions of the locations of the abnormal signals on FLAIR and DWI are presented in Table 4.

**Table 3:** The findings of the first cerebrospinal fluid (CSF) and electroencephalography (EEG) studies.

	**Autoimmune SE (*n* = 25)**	**Infectious SE (*n* = 21)**	***p*-value**	**OR (95% CI)**
The first CSF findings				
CSF protein (mg/dL)	34.8 (23.5–110.9)	93.0 (38.1–260.9)	0.014	
CSF WBC count (cell/mm^3^)	3 (0–18)	20 (2.5–536)	0.011	
Neutrophilic predominance	2 (15.4)	9 (52.9)	0.034	0.162 (0.03–0.96)
Lymphocytic predominance	11 (84.6)	8 (47.1)	0.034	6.19 (1.04–36.78)
^a^CSF/Blood glucose ratio <0.5	6 (24.0)	9 (56.3)	0.036	0.25 (0.06–0.95)
^b^IgG index >0.6	11 (55.0)	4 (66.7)	0.612	0.61 (0.09–4.14)
The first EEG finding				
Normal	1 (4.0)	3 (14.3)	0.318	0.25 (0.02–2.61)
Background slowing activity	14 (56.0)	6 (28.6)	0.081	0.51 (0.24–1.09)
Sporadic epileptiform discharge	2 (8.0)	4 (19.0)	0.239	2.38 (0.48–11.74)
Periodic discharge	3 (12.0)	3 (14.3)	0.769	1.19 (0.27–5.29)
Electrographic seizure	5 (20.0)	5 (23.8)	0.688	1.19 (0.4–3.56)

*Continuous variables were presented as median (interquartile range)*.

*Categorical variables were presented as n (%)*.

*CI, confidence interval; OR, odds ratio; SE, status epilepticus; WBC, white blood cell*.

a *The CSF/blood glucose ratio was available in 16 patients with infectious and all patients with autoimmune etiology*.

b *The IgG index was available in six patients with infectious and 20 patients with autoimmune etiology*.

**Table 4:** The findings of magnetic resonance imaging study.

	**Autoimmune SE (*n* = 25)**	**Infectious SE (*n* = 21)**	***p*-value**	**OR (95% CI)**
FLAIR and T2 abnormalities	11 (44)	8 (38.1)	0.685	1.28 (0.39–4.17)
Lateralization				
Unilateral	5 (20.0)	3 (14.3)	0.729	0.72 (0.11–4.62)
Bilateral	6 (24.0)	5 (23.8)		
Location				
Temporal lobe	9 (36)	5 (23.8)		
Mesial temporal lobe	7 (28)	4 (19)		
Lateral temporal lobe	2 (8)	1 (4.8)		
Frontal lobe	6 (24)	3 (14.3)		
Parietal lobe	7 (28)	2 (9.5)		
Occipital lobe	7 (28)	4 (19)		
Basal ganglion	0 (0)	2 (9.5)		
Multiple lobes	7 (28)	3 (14.3)	0.367	2.10 (0.41–10.66)
DWI abnormalities	14 (56)	10 (50)	0.688	1.27 (0.39–4.14)
Lateralization				
Unilateral	5 (35.7)	6 (60)	0.408	0.37 (0.07–1.97)
Bilateral	9 (64.3)	4 (40)	0.408	2.7 (0.51–14.37)
Location				
Temporal lobe	11 (78.6)	7 (70)		
Mesial temporal lobe	7 (50)	3 (30)		
Lateral temporal lobe	4 (28.6)	5 (50)		
Frontal lobe	6 (42.9)	5 (50)		
Parietal lobe	6 (42.9)	5 (50)		
Occipital lobe	5 (35.7)	5 (50)		
Basal ganglion	0 (0)	4 (40)		
Multiple lobes	11 (78.6)	6 (60)	0.393	2.44 (0.41–14.75)

Categorical variables were presented as n (%).

*CI, confidence interval; DWI, diffusion-weighted imaging; FLAIR, fluid-attenuated inversion recovery; OR, odds ratio; SE, status epilepticus*.

## Discussion

Inflammatory SE is a previously under-recognized subgroup of SE. In the current study, 9.2% of all cases of SE were related to an inflammatory etiology, which is in accordance with previous studies (range from 6 to 12.8%) (11, 20). Inflammatory SE has two main etiologies, infectious, and autoimmune SE, which is at times difficult to differentiate at the initial presentation. We found that younger age, female sex, the presence of psychosis, non-convulsive SE, lymphocytic predominance in CSF were more commonly observed in the patients with autoimmune SE, while a high CSF total protein level, pleocytosis, and reduced glucose ratio were more common in those with infectious SE. EEG and MRI are important tools to confirm the diagnosis of SE and exclude structural lesions (19, 28), but were not particularly helpful in the current study.

Among all patients with SE, infection accounted for 4.2% and autoimmune accounted for 5%. This suggests that autoimmune SE is as common as infectious SE (20, 29), and therefore clinical features that can distinguish the two entities are important for intensive care physicians who care for patients with SE. We observed some differences in the presenting features of those with autoimmune and infectious SE. The age at onset was younger in the patients with autoimmune SE, which has also been reported in two previous studies (11, 20). Female predominance was also observed in the autoimmune SE group in this study, which is in accordance with previous reports that reported females predominance in autoimmune encephalitis and systemic autoimmune disorders (14, 30, 31).

The onset symptoms of autoimmune SE can be various. Alteration in mental status is the cardinal symptom (12), but provides little information about the underlying etiology. In our patients, the presenting symptoms of inflammatory SE included fever, decreased consciousness, seizure, upper respiratory tract infection, headache, psychosis, fatigue, and cognitive decline. Of note, psychosis was present only in those with autoimmune SE and not in those with infectious SE. Other studies have also reported that psychosis is the dominant presenting symptom among patients with autoimmune encephalitis (14, 29, 32, 33). In addition, we found that more of the patients with autoimmune SE had non-convulsive SE compared to those with infectious SE, which was not reported in the two previous studies (11, 20). This may be due to the difficulty in recognizing non-convulsive SE clinically without EEG monitoring or because it was not specifically looked for. Super refractory SE was also more prevalent in the autoimmune SE group, which may be due to the difficulty in making a diagnosis and the ineffectiveness of traditional SE treatment to control seizure activity without immunotherapy (34). When non-convulsive SE or psychosis followed by SE occurs in patients with a young age and female sex, autoimmune SE should be considered.

CSF studies are an important tool to identify the cause of SE, however, such studies can be challenging clinically. Neutrophilic predominant pleocytosis usually points toward a bacterial infection or the early stage of viral encephalitis, especially in the first 24 to 48 hours (24). Lymphocytic predominant pleocytosis was associated with autoimmune SE in our study, but it was also often seen in cases of viral encephalitis-related SE (17, 24). Intensive care physicians often face a dilemma over whether to use antiviral therapy or immunotherapy when the diagnosis is unclear. Other parameters of the CSF can aid in the differential diagnosis, as our data suggested that the patients with an infectious etiology usually had a higher CSF protein level, although prolonged SE itself may result in a milder elevation of lactate and/or total protein levels. This was also reported by Oyanguren et al. who found similar white blood cell count between patients with viral infections and autoimmune processes, but that the protein level was higher in those with a CNS viral infection (35). Therefore, a high protein level in patients with lymphocytic predominance pleocytosis may suggest a viral etiology.

MRI can aid in the search for the etiology of SE, but with limitations. Limbic encephalitis may present as an increased FLAIR/T2 signal or abnormal DWI in the medial temporal lobes (36–38), and it can be used in helping to make the diagnosis of autoimmune encephalitis (16). Prolonged SE itself can also cause similar changes to some viral infectious in MRI signal with DWI abnormalities in the hippocampus and pulvinar (39), particularly herpes simplex encephalitis (18). Furthermore, these MRI patterns may not be present in all types of autoimmune SE and one study reported that 60% of the MRI findings in patients with anti-NMDA encephalitis may have been normal (14). Our data showed that no specific MRI findings could differentiate autoimmune and infectious SE.

EEG is routinely used to evaluate patients with seizures or disturbed consciousness. Slow background activity was more dominant in autoimmune patients compared with other etiologies of seizure (40), although we found no statistical difference in EEG findings between the two groups. Our study showed that at an early stage of inflammatory SE, it remains difficult to differentiate the two entities using currently available para-clinical investigations. The early use of auto-antibody assays may be needed when autoimmune SE is suspected clinically.

We found that general anesthesia was more commonly used in the patients with autoimmune SE. This is in accordance with previous studies in which patients with autoimmune SE were less responsive to AEDs (11, 20, 30, 41). The reason why AEDs are less effective for autoimmune SE remains to be clarified, although it is well-known that the treatment of autoimmune SE requires prompt immunotherapy (14, 15), which may then reduce the use of general anesthesia.

The functional outcomes were similar in both infectious and autoimmune groups with a similar mRS score at discharge and similar mortality rate during admission. However, most of our patients had a poor outcome at discharge or last follow-up (63.0 and 65.2%, respectively). Our study showed that the predictive values of STESS and END-IT scores were not in the same direction. That is, STESS was more sensitive but END-IT was more specific in terms of predicting the outcomes at discharge. More studies may be needed to compare the use of these two scores. In addition to functional impairments, a recent study reported that patients also had substantial impairments in their quality of life after SE (42). Our patients with autoimmune SE had a mortality rate of 20%, which is similar to other studies ranging from 10 to 23% (20, 30, 43). A recent population-based study conducted in Germany reported a hospital mortality rate for all types of SE of 14.8% with a higher rate in those with refractory SE and super refractory SE (15.0 and 39.9%, respectively) (44). The higher mortality rate in patients with autoimmune etiology compared to those with all-cause SE may be related to the high percentage of super refractory SE among patients with an autoimmune etiology. This higher mortality rate compared to all-cause SE emphasize the need for rapid recognition of the condition and prompt treatment toward the underlying causes in addition to standard SE care.

The limitations of this study are that it was conducted at a single hospital and that the design was retrospective. In addition, the study was started before the availability of recent autoimmune encephalitis screening tests and immunotherapies, which may have affected the outcomes.

In conclusion, we observed that patients with autoimmune SE had a younger age at onset, female predominance, and often presented with psychosis, super-refractory SE and non-convulsive SE. The initial clinical investigations including EEG and MRI only provided limited information about the underlying etiology. CSF tests were helpful in diagnosing bacterial infectious-related SE but had difficulty in differentiating viral encephalitis and autoimmune SE. Since these two etiologies have different treatment strategies and the presenting symptoms are quite similar (12, 29), it is important to differentiate the two conditions as soon as possible. The patient characteristics and presenting features identified in our study may provide clinicians with some clues about the underlying etiology. Empiric treatment can be given based on these clinical clues while waiting for the results of more definitive diagnostic tests such as viral serology tests and neuronal surface auto-antibody screening.

## Ethics Statement

This study is approved by the Chang Gung Medical Foundation Institutional Review Board (IRB No.: 103-3665B and 201800677B0).

## Author Contributions

All authors have read and approved the final manuscript. C-HL contributed to clinical data analysis and draft of the manuscript. Y-TL, C-JH, and F-YS had contributions to clinical data acquisition and analysis. M-HT had substantial contributions to the conception and design of the study, data analysis, critical revision, and final approval of the manuscript.

### Conflict of Interest Statement

The authors declare that the research was conducted in the absence of any commercial or financial relationships that could be construed as a potential conflict of interest.

## References

[1] Bermeo-Ovalle A Bleck T. Status epilepticus in the intensive care unit. Semin Neurol. (2016) 36:550–9. 10.1055/s-0036-1592357 27907959

[2] Wu YW Shek DW Garcia PA Zhao S Johnston SC. Incidence and mortality of generalized convulsive status epilepticus in California. Neurology (2002) 58:1070–6. 10.1212/WNL.58.7.1070 11940695

[3] Lv RJ Wang Q Cui T Zhu F Shao XQ. Status epilepticus-related etiology, incidence and mortality: a meta-analysis. Epilepsy Res. (2017) 136:12–7. 10.1016/j.eplepsyres.2017.07.006 28734267

[4] Glauser T Shinnar S Gloss D Alldredge B Arya R Bainbridge J. Evidence-based guideline: treatment of convulsive status epilepticus in children and adults: report of the guideline committee of the American epilepsy society. Epilepsy Curr. (2016) 16:48–61. 10.5698/1535-7597-16.1.48 26900382 PMC4749120

[5] Jagoda A Riggio S. Refractory status epilepticus in adults. Ann Emerg Med. (1993) 22:1337–48. 10.1016/S0196-0644(05)80120-9 8333641

[6] Tsai MH Chuang YC Chang HW Chang WN Lai SL Huang CR. Factors predictive of outcome in patients with de novo status epilepticus. QJM (2009) 102:57–62. 10.1093/qjmed/hcn149 19015144

[7] Koubeissi M Alshekhlee A. In-hospital mortality of generalized convulsive status epilepticus: a large US sample. Neurology (2007) 69:886–93. 10.1212/01.wnl.0000269791.96189.70 17724291

[8] Rossetti AO Hurwitz S Logroscino G Bromfield EB. Prognosis of status epilepticus: role of aetiology, age, and consciousness impairment at presentation. J Neurol Neurosurg Psychiatry (2006) 77:611–5. 10.1136/jnnp.2005.080887 16614020 PMC2117456

[9] Seinfeld S Goodkin HP Shinnar S. Status epilepticus. Cold Spring Harb perspect Med. (2016) 6:a022830. 10.1101/cshperspect.a022830 26931807 PMC4772080

[10] Singh TD Fugate JE Rabinstein AA. The spectrum of acute encephalitis: causes, management, and predictors of outcome. Neurology (2015) 84:359–66. 10.1212/WNL.0000000000001190 25540320

[11] Shin JW Koo YS Kim YS Kim DW Kim KK Lee SY. Clinical characterization of unknown/cryptogenic status epilepticus suspected as encephalitis: a multicenter cohort study. J Neuroimmunol. (2018) 315:1–8. 10.1016/j.jneuroim.2017.12.004 29306399

[12] Venkatesan A Tunkel AR Bloch KC Lauring AS Sejvar J Bitnun A. Case definitions, diagnostic algorithms, and priorities in encephalitis: consensus statement of the international encephalitis consortium. Clin Infect Dis. (2013) 57:1114–28. 10.1093/cid/cit458 23861361 PMC3783060

[13] Suthar R Sankhyan N. Bacterial Infections of the central nervous system. Indian J Pediatr. (2018) 86:60–9. 10.1007/s12098-017-2477-z 29297142

[14] Titulaer MJ McCracken L Gabilondo I Armangue T Glaser C Iizuka T. Treatment and prognostic factors for long-term outcome in patients with anti-NMDA receptor encephalitis: an observational cohort study. Lancet Neurol. (2013) 12:157–65. 10.1016/S1474-4422(12)70310-1 23290630 PMC3563251

[15] Shin YW Lee ST Park KI Jung KH Jung KY Lee SK. Treatment strategies for autoimmune encephalitis. Ther Adv Neurol Disord. (2018) 11:1756285617722347. 10.1177/1756285617722347 29399043 PMC5784571

[16] Graus F Titulaer MJ Balu R Benseler S Bien CG Cellucci T. A clinical approach to diagnosis of autoimmune encephalitis. Lancet Neurol. (2016) 15:391–404. 10.1016/S1474-4422(15)00401-9 26906964 PMC5066574

[17] Armangue T Leypoldt F Dalmau J. Autoimmune encephalitis as differential diagnosis of infectious encephalitis. Curr Opin Neurol. (2014) 27:361–8. 10.1097/WCO.0000000000000087 24792345 PMC4132825

[18] Sureka J Jakkani RK. Clinico-radiological spectrum of bilateral temporal lobe hyperintensity: a retrospective review. Br J Radiol. (2012) 85:e782–92. 10.1259/bjr/30039090 22422381 PMC3487100

[19] Lancaster E. The diagnosis and treatment of autoimmune encephalitis. J Clin Neurol. (2016) 12:1–13. 10.3988/jcn.2016.12.1.1 26754777 PMC4712273

[20] Spatola M Novy J Du Pasquier R Dalmau J Rossetti AO. Status epilepticus of inflammatory etiology: a cohort study. Neurology (2015) 85:464–70. 10.1212/WNL.0000000000001717 26092915 PMC4534074

[21] Brophy GM Bell R Claassen J Alldredge B Bleck TP Glauser T. Guidelines for the evaluation and management of status epilepticus. Neurocrit Care (2012) 17:3–23. 10.1007/s12028-012-9695-z 22528274

[22] Ferlisi M Shorvon S. The outcome of therapies in refractory and super-refractory convulsive status epilepticus and recommendations for therapy. Brain (2012) 135(Pt 8):2314–28. 10.1093/brain/aws091 22577217

[23] Trinka E Cock H Hesdorffer D Rossetti AO Scheffer IE Shinnar S. A definition and classification of status epilepticus–Report of the ILAE task force on classification of status epilepticus. Epilepsia (2015) 56:1515–23. 10.1111/epi.13121 26336950

[24] Tuppeny M. Viral meningitis and encephalitis. Crit Care Nurs Clin North Am. (2013) 25:363–80. 10.1016/j.ccell.2013.04.003 23981453

[25] Rossetti AO Logroscino G Milligan TA Michaelides C Ruffieux C Bromfield EB. Status Epilepticus Severity Score (STESS): a tool to orient early treatment strategy. J Neurol. (2008) 255:1561–6. 10.1007/s00415-008-0989-1 18769858

[26] Gao Q Ou-Yang TP Sun XL Yang F Wu C Kang T. Prediction of functional outcome in patients with convulsive status epilepticus: the END-IT score. Crit Care (2016) 20:46. 10.1186/s13054-016-1221-9 26916702 PMC4768332

[27] Hirsch LJ LaRoche SM Gaspard N Gerard E Svoronos A Herman ST. American clinical neurophysiology society's standardized critical care EEG terminology: 2012 version. J Clin Neurophysiol. (2013) 30:1–27. 10.1097/WNP.0b013e3182784729 23377439

[28] Heine J Pruss H Bartsch T Ploner CJ Paul F Finke C. Imaging of autoimmune encephalitis–Relevance for clinical practice and hippocampal function. Neuroscience (2015) 309:68–83. 10.1016/j.neuroscience.2015.05.037 26012492

[29] Saraya A Mahavihakanont A Shuangshoti S Sittidetboripat N Deesudchit T Callahan M. Autoimmune causes of encephalitis syndrome in Thailand: prospective study of 103 patients. BMC Neurol. (2013) 13:150. 10.1186/1471-2377-13-150 24139084 PMC3853593

[30] Holzer FJ Rossetti AO Heritier-Barras AC Zumsteg D Roebling R Huber R. Antibody-mediated status epilepticus: a retrospective multicenter survey. Eur Neurol. (2012) 68:310–7. 10.1159/000341143 23051892

[31] Gubbels Bupp MR Potluri T Fink AL Klein SL. The confluence of sex hormones and aging on immunity. Front Immunol. (2018) 9:1269. 10.3389/fimmu.2018.01269 29915601 PMC5994698

[32] Hao Q Wang D Guo L Zhang B. Clinical characterization of autoimmune encephalitis and psychosis. Compr Psychiatry (2017) 74:9–14. 10.1016/j.comppsych.2016.12.006 28081431

[33] Lim JA Lee ST Jung KH Kim S Shin JW Moon J. Anti-N-methyl-d-aspartate receptor encephalitis in Korea: clinical features, treatment, and outcome. J Clin Neurol. (2014) 10:157–61. 10.3988/jcn.2014.10.2.157 24829602 PMC4017019

[34] Varley J Taylor J Irani SR. Autoantibody-mediated diseases of the CNS: structure, dysfunction and therapy. Neuropharmacology (2018) 132:71–82. 10.1016/j.neuropharm.2017.04.046 28476644

[35] Oyanguren B Sanchez V Gonzalez FJ de Felipe A Esteban L Lopez-Sendon JL. Limbic encephalitis: a clinical-radiological comparison between herpetic and autoimmune etiologies. Eur J Neurol. (2013) 20:1566–70. 10.1111/ene.12249 23941332

[36] Lai M Hughes EG Peng X Zhou L Gleichman AJ Shu H. AMPA receptor antibodies in limbic encephalitis alter synaptic receptor location. Ann Neurol. (2009) 65:424–34. 10.1002/ana.21589 19338055 PMC2677127

[37] Lai M Huijbers MG Lancaster E Graus F Bataller L Balice-Gordon R. Investigation of LGI1 as the antigen in limbic encephalitis previously attributed to potassium channels: a case series. Lancet Neurol. (2010) 9:776–85. 10.1016/S1474-4422(10)70137-X 20580615 PMC3086669

[38] Hoftberger R Titulaer MJ Sabater L Dome B Rozsas A Hegedus B. Encephalitis and GABAB receptor antibodies: novel findings in a new case series of 20 patients. Neurology (2013) 81:1500–6. 10.1212/WNL.0b013e3182a9585f 24068784 PMC3888170

[39] Mendes A Sampaio L. Brain magnetic resonance in status epilepticus: a focused review. Seizure (2016) 38:63–7. 10.1016/j.seizure.2016.04.007 27156207

[40] Limotai C Denlertchaikul C Saraya AW Jirasakuldej S. Predictive values and specificity of electroencephalographic findings in autoimmune encephalitis diagnosis. Epilepsy Behav. (2018) 84:29–36. 10.1016/j.yebeh.2018.04.007 29738958

[41] Dubey D Blackburn K Greenberg B Stuve O Vernino S. Diagnostic and therapeutic strategies for management of autoimmune encephalopathies. Expert Rev Neurother. (2016) 16:937–49. 10.1080/14737175.2016.1189328 27171736

[42] Kortland LM Knake S von Podewils F Rosenow F Strzelczyk A. Socioeconomic outcome and quality of life in adults after status epilepticus: a multicenter, longitudinal, matched case-control analysis from Germany. Front Neurol. (2017) 8:507. 10.3389/fneur.2017.00507 29018404 PMC5622933

[43] Holzer FJ Seeck M Korff CM. Autoimmunity and inflammation in status epilepticus: from concepts to therapies. Expert Rev Neurother. (2014) 14:1181–202. 10.1586/14737175.2014.956457 25201402

[44] Strzelczyk A Ansorge S Hapfelmeier J Bonthapally V Erder MH Rosenow F. Costs, length of stay, and mortality of super-refractory status epilepticus: a population-based study from Germany. Epilepsia (2017) 58:1533–41. 10.1111/epi.13837 28681418

